# EWSR1 as a regulatory target in hepatic stellate cell apoptosis and liver fibrosis therapy

**DOI:** 10.1016/j.bbrep.2026.102526

**Published:** 2026-02-27

**Authors:** Zhang Shiwan, Luo Guangcheng, Maisarah Abdul Mutalib

**Affiliations:** a Department of Infectious Diseases, Affiliated Hospital, Clinical Medical School, North Sichuan Medical College, Nanchong, Sichuan, China; b Department of Clinical Laboratory, Affiliated Hospital of North Sichuan Medical College, Nanchong, Sichuan, China; c School of Graduate Studies, Postgraduate Centre, Management and Science University, University Drive, Shah Alam, Selangor, Malaysia; d International Medical School, Management and Science University, University Drive, Shah Alam, Selangor, Malaysia

**Keywords:** Hepatic stellate cells, Extracellular matrix accumulation, TGF-β/SMAD signaling, Oxidative stress regulation, Liver fibrosis, RNA-Binding proteins, Anti-fibrotic therapeutic targets

## Abstract

Liver fibrosis is a progressive condition driven by hepatic stellate cell (HSC) activation and resistance to apoptosis, culminating in excessive extracellular matrix (ECM) accumulation and organ dysfunction. Current antifibrotic therapies remain limited, as most target broad pathways such as TGF-β signaling or oxidative stress without addressing upstream regulators. Emerging evidence identifies the RNA-binding protein Ewing Sarcoma Breakpoint Region 1 (EWSR1) as a pivotal modulator of HSC fate. Through transcriptional regulation, non-coding RNA networks, and stress-granule dynamics, EWSR1 integrates TGF-β signaling, oxidative stress responses, and apoptosis resistance. Inhibition of EWSR1 has been reported in experimental models to suppress fibrosis-related gene expression and restore apoptotic sensitivity in activated HSCs, highlighting its therapeutic potential. This review critically synthesizes recent insights into EWSR1 biology, its crosstalk with profibrotic pathways, and its regulatory influence on HSC activation. We further compare EWSR1 with conventional antifibrotic approaches, outline research gaps, and propose directions for translational development. By positioning EWSR1 as a novel molecular node in fibrogenesis, this article underscores its promise as a next-generation therapeutic target for halting or reversing liver fibrosis.

## Highlights

• EWSR1 is identified as a novel regulator in hepatic stellate cell activation.
• EWSR1 modulates fibrosis via transcriptional and non-coding RNA mechanisms.
• Suppression of EWSR1 restores apoptosis sensitivity in activated HSCs.
• Integrates EWSR1 signaling with TGF-β and oxidative stress pathways.
• Positions EWSR1 as a potential antifibrotic therapeutic target.

## 1 Introduction

Liver fibrosis is a major pathological outcome of chronic liver injury, defined by excessive extracellular matrix (ECM) deposition that gradually replaces functional liver parenchyma and compromises organ function (Henderson et al., 2020). It arises from multiple etiologies including viral hepatitis, alcoholic liver disease, and the increasingly prevalent non-alcoholic fatty liver disease (NAFLD) all of which converge on sustained oxidative stress and inflammatory signaling [1]. These insults perpetuate hepatocellular damage, stimulate immune cell recruitment, and trigger hepatic stellate cell (HSC) activation, the central driver of fibrogenesis.

Globally, viral hepatitis B and C remain dominant contributors to liver fibrosis, where chronic immune activation exacerbates hepatocellular injury and perpetuates HSC stimulation [2]. In parallel, the surge in obesity and metabolic syndrome has positioned NAFLD as an emerging global driver, particularly in Western and developing nations. NAFLD is mechanistically tied to insulin resistance, dyslipidemia, and lipotoxicity, which amplify oxidative stress and apoptosis in hepatocytes, reinforcing the fibrotic cascade [3].

These pathogenic drivers expose the liver to recurrent cycles of inflammation, apoptosis, and regeneration, which collectively accelerate fibrotic remodeling. This process is orchestrated through dynamic interactions among hepatocytes, Kupffer cells, and particularly HSCs—the principal effector cells of fibrosis [4]. Normally quiescent and vitamin A–storing, HSCs adopt a myofibroblast-like phenotype upon injury, characterized by proliferation, migration, and secretion of fibrillar collagens (types I and III) that progressively remodel hepatic architecture [5].

The expansion of activated HSC populations accelerates ECM accumulation, leading to septa formation, lobular distortion, and progressive hepatic dysfunction [6]. Activated HSCs also secrete pro-fibrotic mediators including transforming growth factor-β (TGF-β) and platelet-derived growth factor (PDGF), which not only sustain their own activation but also recruit macrophages and other immune cells, thereby reinforcing a pro-inflammatory and pro-fibrotic microenvironment [7].

Given this central role, fibrosis regression fundamentally depends on restraining HSC activation and restoring their apoptotic clearance. Indeed, induction of apoptosis in activated HSCs facilitates ECM degradation and structural remodeling, offering a potential avenue for fibrosis reversal [8]. Accordingly, the regulation of HSC activation–apoptosis balance has become a cornerstone of antifibrotic research.

Contemporary antifibrotic strategies have largely concentrated on modulating TGF-β signaling or oxidative stress to influence HSC apoptosis, yet no approved therapy effectively halts or reverses fibrosis. This therapeutic gap underscores the need for novel, mechanism-based targets. Recent evidence implicates the RNA-binding protein EWSR1 as a previously unrecognized regulator of fibrotic signaling, although its precise molecular roles remain to be fully defined.

This review aims to consolidate current knowledge on the emerging role of EWSR1 in HSC biology and liver fibrosis. Specifically, we explore how EWSR1 intersects with TGF-β signaling and oxidative stress responses to modulate HSC apoptosis and activation. We critically analyze its potential as a therapeutic target, highlight unresolved mechanistic questions, and outline avenues for future research. By synthesizing this evidence, the review positions EWSR1 as a novel molecular node in fibrogenesis with translational potential for antifibrotic therapy.

## 2 HSCs in fibrosis

HSCs are mesenchymal cells residing in the space of Disse, where they normally function as vitamin A reservoirs. In response to chronic liver injury, HSCs transdifferentiate into myofibroblast-like cells, acquiring proliferative and profibrotic properties that establish them as the central drivers of fibrosis [9].

After liver injury, pro-inflammatory cytokines are released by inflammatory cells (such as TGF-β, PDGF, and IL-6), along with ROS, work together to induce HSCs activation. Activated HSCs undergo a series of significant phenotypic changes, including proliferation, migration, and the synthesis and secretion of large amounts of ECM proteins and pro-fibrotic factors [7]. These characteristics make HSCs an irreversible driving force in the fibrosis process.

The phenotypic transition of activated HSCs typically involves several key molecules and signaling pathways. The TGF-β signaling pathway is one of the primary drivers of HSCs activation, upregulated during liver injury. It activates downstream fibrosis-related genes through mothers against decapentaplegic homolog (SMAD) family proteins, promoting ECM synthesis [10]. Additionally, platelet-derived growth factor (PDGF) activates the phosphoinositide 3-kinase (PI3K)/protein kinase B (Akt) and ERK signaling pathways via its receptor, further promoting HSCs proliferation and migration [11]. The interplay of these signaling pathways not only facilitates HSCs activation but also contributes to the formation of a fibrotic microenvironment, exacerbating liver injury and fibrosis progression.

The activation of HSCs is an irreversible process. Once the activated state is established, HSCs self-sustain and produce large amounts of pro-fibrotic factors, creating a persistent fibrotic microenvironment in the liver. This environment is rich in ECM proteins, cytokines, and chemokines, continuously recruiting immune cells and other fibroblasts, forming a complex cell-cell interaction network that makes liver fibrosis difficult to reverse [12].

**Fig. 1:**
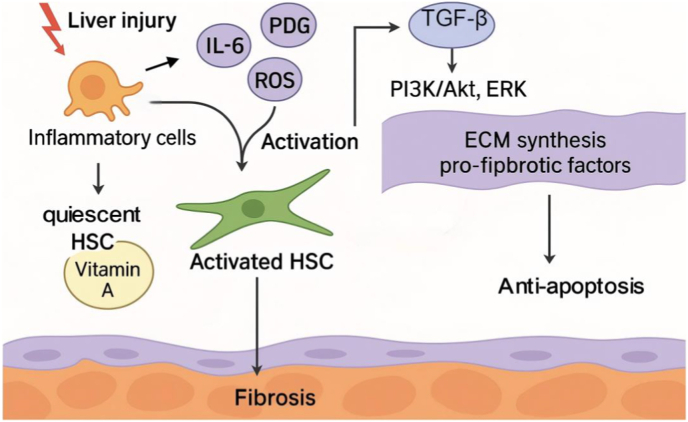
Schematic diagram illustrating the role of HSCs in liver fibrosis. Quiescent HSCs, located in the space of Disse and storing vitamin A, are activated by chronic liver injury. Pro-inflammatory cytokines (IL-6, TNF-α), PDGF, and ROS released from inflammatory cells, together with TGF-β signaling via SMAD3 and PI3K/Akt pathways, drive HSCs activation. Activated HSCs undergo proliferation, migration, and ECM synthesis, producing pro-fibrotic factors and secreting tissue inhibitors of TIMPs to inhibit ECM degradation, leading to progressive fibrosis. Persistent activation also resists apoptosis, sustaining a fibrotic microenvironment.

Additionally, HSCs secrete various tissue inhibitors of metalloproteinases (such as TIMP-1), which hinder ECM degradation and further exacerbate fibrotic deposition [13]. This persistent microenvironment, enriched with ECM proteins, cytokines, and tissue inhibitors of metalloproteinases (TIMPs), stabilizes fibrosis and makes it difficult to reverse (Fig. 1). This fibrotic microenvironment has a profound impact on the normal structure and function of liver tissue. The excessive accumulation of ECM not only increases liver stiffness but also disrupts the lobular architecture, impairing blood flow and nutrient distribution, ultimately leading to progressive liver function decline [14].

Recent studies have shown that inducing HSCs apoptosis is a key strategy for reversing fibrosis. If activated HSCs are not cleared or reverted, they will continue to secrete fibrotic proteins, exacerbating fibrosis. Therefore, promoting HSCs apoptosis can slow down or partially reverse the fibrotic process. For example, certain anti-fibrotic drugs alleviate fibrosis by activating apoptotic pathways in HSCs or inhibiting their proliferation[8].

In the process of fibrosis reversal, the clearance of activated HSCs promotes ECM degradation and the remodeling of fibrotic structures. Some studies have found that inducing HSCs apoptosis by specifically regulating apoptotic signaling pathways, such as Fas/FasL, p53, and Bcl-2/Bax, can help alleviate fibrosis symptoms [15]. However, the regulatory mechanisms of HSCs apoptosis are highly complex and influenced by multiple signaling pathways, making the identification of effective and precise therapeutic targets a key focus of ongoing research.

Due to the central role of HSCs in liver fibrosis, regulating their activation and apoptosis has become a key strategy for anti-fibrotic therapy. Although current anti-fibrotic drugs have yet to achieve optimal therapeutic outcomes, targeting HSCs provides new directions for drug development [16]. Recently, attention has shifted toward RNA-binding proteins as novel regulators of HSC fate. In particular, EWSR1 has emerged as a potential molecular link between TGF-β signaling, oxidative stress, and apoptosis resistance in HSCs. Although mechanistic details are still unfolding, preliminary evidence supports its candidacy as a promising antifibrotic target [17].

## 3 Role of the TGF-β signaling pathway in liver fibrosis

The TGF-β signaling pathway is one of the key pathways regulating liver fibrosis progression. By activating downstream SMAD family proteins and other non-SMAD pathways, TGF-β drives HSCs activation, ECM synthesis, and fibrosis progression. TGF-β not only plays a central role in HSCs activation but also acts as a key regulator in maintaining and advancing the fibrotic microenvironment [18].

As a multifunctional cytokine, TGF-β is secreted at increased levels in liver-injured tissues and serves as a major driver of HSC activation. Upon binding to its cell membrane receptors, TGF-β triggers a receptor-type protein kinase phosphorylation cascade, leading to the activation of intracellular SMAD proteins (primarily SMAD2 and SMAD3). These activated SMAD proteins form a complex that translocates into the nucleus to regulate the expression of fibrosis-related genes [19]. In HSCs, the TGF-β/SMAD signaling pathway upregulates a series of profibrotic genes, such as collagen, fibronectin, and tissue inhibitors of metalloproteinases (e.g., TIMP-1), thereby promoting ECM accumulation and fibrosis formation [20].

In addition to the SMAD pathway, TGF-β also exerts its effects through non-SMAD signaling. For instance, TGF-β can activate pathways such as mitogen-activated protein kinase (MAPK), PI3K/Akt, and Rho GTPases, which promote HSCs proliferation, migration, and resistance to apoptosis. These non-SMAD pathways exhibit crucial synergistic effects under different cellular and microenvironmental stimuli, endowing TGF-β signaling with high plasticity and adaptability [21].

During liver fibrosis progression, persistent TGF-β activation drives HSCs proliferation and phenotypic transformation. Activated HSCs exhibit a high level of synthetic activity, producing large amounts of ECM proteins, cytokines, and chemokines. These secreted factors not only sustain HSCs activation but also recruit and activate other cell types, such as macrophages and lymphocytes, creating a highly profibrotic microenvironment [22].

TGF-β signaling in HSCs is not limited to gene expression regulation; it also significantly inhibits apoptosis. Activated HSCs, through TGF-β signaling, upregulate the expression of anti-apoptotic protein Bcl-2 while downregulating the pro-apoptotic factor Bax, thereby reducing their apoptotic tendency [22]. The anti-apoptotic nature of HSCs further exacerbates fibrosis progression, making it difficult to reverse [8].

Within the fibrotic microenvironment, TGF-β not only directly acts on HSCs but also maintains fibrosis progression through interactions with other cell types. For example, TGF-β promotes the polarization of macrophages toward the M2 phenotype, enhancing their profibrotic effects and exacerbating HSCs activation. Additionally, TGF-β induces endothelial and fibroblast transdifferentiation, facilitating angiogenesis and fibrotic tissue remodeling [23]. The interplay between TGF-β and these cell types further reinforces the persistence and irreversibility of the fibrotic microenvironment.

During liver injury, TGF-β and oxidative stress often act together to amplify the fibrotic response. Reactive oxygen species (ROS) generated by oxidative stress not only cause direct hepatocyte damage but also increase TGF-β expression and activity, thereby amplifying its signaling pathway effects [24]. Studies have shown that ROS can enhance TGF-β/SMAD pathway profibrotic effects by oxidatively modifying SMAD3, increasing its binding affinity to TGF-β receptors [25].

**Fig. 2:**
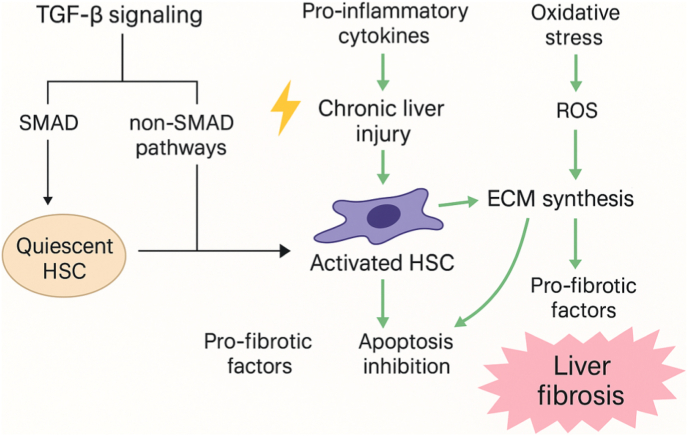
Schematic diagram illustrates the roles of TGF-β signaling and oxidative stress in liver fibrosis. TGF-β activates both SMAD and non-SMAD pathways, driving quiescent HSCs toward activation, ECM synthesis, and apoptosis resistance. Chronic liver injury and pro-inflammatory cytokines further promote HSCs activation. Oxidative stress enhances ROS production, which stimulates ECM deposition and amplifies TGF-β signaling, creating a positive feedback loop that sustains fibrosis progression.

Moreover, TGF-β downregulates antioxidant enzymes such as glutathione peroxidase and superoxide dismutase, exposing liver tissue to higher oxidative stress levels and further promoting HSCs activation. This interaction between TGF-β and ROS forms a positive feedback loop that accelerates fibrosis progression and renders it more irreversible [26]. This reciprocal reinforcement between oxidative stress and TGF-β signaling forms a vicious cycle that accelerates fibrosis (Fig. 2).

Given the central role of TGF-β signaling in liver fibrosis, it has become a primary target for antifibrotic therapy. However, due to TGF-β′s pleiotropic effects and widespread expression in multiple organs, direct inhibition may lead to adverse effects such as immunosuppression and cancer. Consequently, researchers are exploring targeted interventions downstream of TGF-β, such as SMAD3 or non-SMAD pathways, to reduce side effects and improve therapeutic specificity [14].

In recent years, the RNA-binding protein EWSR1 has been found to potentially influence the regulation of the TGF-β signaling pathway [27]. EWSR1 not only modulates TGF-β transcriptional processes but may also participate in oxidative stress regulation, thereby exerting potential antifibrotic effects in fibrosis progression [17]. This discovery provides a new research direction for antifibrotic therapy, suggesting that modulating EWSR1 to indirectly influence TGF-β signaling may be a promising therapeutic strategy.

## 4 The role of oxidative stress in liver fibrosis

Oxidative stress refers to cellular damage caused when the production of ROS exceeds the cell's antioxidant defense capacity. In the liver, oxidative stress is not only a critical trigger for liver fibrosis but also interacts with multiple signaling pathways to exacerbate fibrosis progression. ROS contribute to fibrosis through various mechanisms, including inducing inflammatory responses, damaging hepatocytes, and activating HSCs [28].

Under normal conditions, ROS act as signaling molecules regulating cell proliferation, differentiation, and metabolism. However, in chronic liver injury caused by factors such as alcohol, viral infections, or fat accumulation, ROS production increases significantly, leading to elevated oxidative stress levels. ROS, including superoxide anion (O_2_^−^), hydrogen peroxide (H_2_O_2_), and hydroxyl radicals (·OH), can directly damage hepatocytes by oxidizing lipids, proteins, and DNA, thus triggering inflammation and cell death [29]. In experimental models, modulation of oxidative stress markers has been shown to influence downstream apoptotic pathways and tissue injury responses [30], reinforcing the concept that redox homeostasis represents a critical regulatory axis in fibrogenic progression.

During liver fibrosis, oxidative stress affects HSCs activation and proliferation through multiple pathways. For instance, ROS activate the MAPK signaling pathway, including ERK, JNK, and p38, promoting HSCs activation and migration[31]. Oxidative stress also upregulates TGF-β expression and enhances its signaling activity, accelerating the formation of pro-fibrotic HSCs phenotypes [26].

In the early stages of fibrosis, oxidative stress promotes HSCs activation both directly and indirectly. ROS can directly activate HSCs through oxidative protein modifications or indirectly by regulating the expression of various cytokines. For example, oxidative stress activates nuclear factor kappa B (NF-κB), leading to increased expression of pro-inflammatory and pro-fibrotic factors such as IL-1β, IL-6, and TGF-β [31]. These factors collectively contribute to the transition of HSCs from a quiescent to an activated state.

During fibrosis progression, oxidative stress contributes to the formation of a pro-inflammatory and pro-fibrotic microenvironment, leading to significant structural changes in liver tissue. Accumulated ROS not only activate HSCs but also stimulate Kupffer cells, liver endothelial cells, and fibroblasts, inducing them to secrete additional pro-inflammatory and pro-fibrotic factors. This positive feedback loop further stabilizes and sustains the fibrotic microenvironment, making fibrosis difficult to reverse [14].

Oxidative stress also disrupts the balance between matrix metalloproteinases (MMPs) and their inhibitors (TIMPs), affecting ECM degradation and accumulation [32]. Under oxidative stress conditions, MMP activity decreases while TIMP-1 expression increases, inhibiting ECM degradation and promoting the persistence of the fibrotic microenvironment. This imbalance leads to continuous ECM deposition in the liver, ultimately rendering fibrosis irreversible.

In addition to promoting HSCs activation, oxidative stress also affects their apoptosis [26]. The apoptosis of HSCs is essential for fibrosis regression; however, oxidative stress upregulates anti-apoptotic proteins such as Bcl-2 while downregulating pro-apoptotic factors such as Bax, thereby inhibiting HSCs apoptosis [33]. This inhibition allows activated HSCs to persist, maintaining their pro-fibrotic phenotype. Mechanistic investigations into methoxyflavone analogs have further demonstrated regulation of mitochondrial apoptotic signaling pathways, including modulation of the Bcl-2/Bax axis and caspase activation [34].

Given the crucial role of oxidative stress in liver fibrosis, regulating oxidative stress levels has emerged as a potential antifibrotic strategy. Antioxidants can mitigate liver damage by scavenging ROS or enhancing antioxidant enzyme activity. For example, clinical and experimental studies have demonstrated the antifibrotic effects of antioxidants such as vitamin E and N-acetylcysteine (NAC) [35]. Bioactive flavonoids have demonstrated significant antioxidant capacity and ROS-scavenging activity across multiple disease models, highlighting their potential to modulate oxidative stress–associated signaling pathways [36].

Recently, the RNA-binding protein EWSR1 has been implicated as a modulator of redox homeostasis in fibrosis. By influencing HSC proliferation, apoptosis, and ROS metabolism, EWSR1 shapes oxidative stress responses. Inhibition of EWSR1 has been reported to reduce ROS levels and attenuate TGF-β signaling, positioning it as a promising antifibrotic target [17]. The interplay between TGF-β, ROS, and HSC activation is summarized in Table 1, which highlights their synergistic contribution to fibrosis progression.

**Table 1:** Key mechanism of TGF-β & ROS linked with activation of fibrosis progression.

Vital Mechanism	Role of TGF-β and ROS	The effects of fibrosis progression	Reference
TGF-β/SMAD signaling pathway	TGF-β binds to SMAD protein through its receptor and activates the SMAD2/3 pathway, leading to the accumulation of ECM and promoting the formation of fibrosis. ROS induces TGF-β signaling by inducing its expression and promoting latent TGF-β activation via LAP.	The activated TGF-β/SMAD pathway promotes the activation and proliferation of HSCs, which ultimately accelerates fibrosis.	[37] [38]
Activation of HSCs	TGF-β stimulates HSCs to transform into myofibroblasts, resulting in more collagen and ECM components. ROS enhanced the activation of HSCs and further increased the synthesis of collagen and fibrosis factors.	The activation of HSCs is central to the fibrotic process, promoting the expansion of fibrosis.	[39] [40]
ECM deposition	Upregulation of TGF-β receptors (TGF-βR1/2), CTGF, and SMAD promotes excessive ECM deposition and reduced degradation. ROS regulates ECM turnover by activating MMPs and suppressing TIMPs.	Excessive accumulation of ECM in fibrosis leads to liver sclerosis and destruction of normal liver function.	[41] [42]
Oxidative stress and cell death	TGF-β may reduce oxidative stress by enhancing antioxidant response, but it can also induce cell death by inducing the generation of ROS. ROS oxidizes cellular components, triggering apoptosis or necrosis in HSCs.	ROS-driven HSC death and hepatocyte injury exacerbate fibrotic progression.	[43] [31]
Signal feedback mechanism	The activation of TGF-β promotes the generation of ROS, forming a vicious circle and further activating the TGF-β/SMAD signaling pathway. ROS can enhance the TGF-β signaling pathway in a variety of ways, such as by enhancing the expression of TGF-β receptors or activating related kinases.	ROS and TGF-β promote each other to form a feedback loop of fibrosis, leading to the aggravation of fibrosis.	[41]

## 5 Molecular function of EWSR1 and its fibrosis regulatory potential

### 5.1 Structure and biological function of EWSR1

EWSR1, a key member of the RNA-binding protein family, plays a significant role in Ewing's sarcoma through gene rearrangement. It participates in various cellular processes, including transcriptional regulation, RNA processing, transport, and cellular stress responses, highlighting its multifunctional importance in maintaining cellular homeostasis [44].

The functional structure of EWSR1 consists of an N-terminal glycine-rich region and a C-terminal poly(guanine) region. The C-terminal region is characterized by an RNA recognition motif (RRM), a zinc finger (ZnF) domain, arginine-glycine-glycine (RGG)-rich domains, and a nuclear localization signal (NLS), which collectively enable EWSR1 to interact with DNA, RNA, and proteins. These structural domains allow EWSR1 to regulate gene expression at multiple levels. Specifically, the RGG-rich domains facilitate DNA binding, enhancing EWSR1's transcriptional activity and promoting the expression of target genes [45]. The N-terminal glycine-rich region of EWSR1 provides a robust platform for molecular interactions, enabling it to function as a scaffold for assembling complexes with diverse transcription factors and RNA-binding proteins. This structural feature is essential for coordinating multiple cellular processes through dynamic protein-protein and protein-RNA interactions. The RRM of EWSR1 allows it to specifically bind to target RNA sequences, enabling its participation in key processes such as mRNA splicing, transport, and degradation.

The central molecular role of EWSR1 lies in transcriptional regulation. It can act directly as a transcriptional activator to stimulate gene expression or form multi-protein complexes with transcription factors to modulate promoter activity. For example, EWSR1 interacts with its FET family counterparts, FUS and TAF15, to regulate transcriptional programs [46,47]. These complexes facilitate the recruitment of RNA polymerase II to promoter regions, thereby enhancing gene transcription. In addition, EWSR1 contributes to chromatin remodeling, modulating chromatin accessibility and enabling transcription factors to more effectively engage with DNA.

EWSR1 acts as a multifunctional protein with essential roles in maintaining cellular homeostasis and adapting to physiological demands. Through interactions with transcription factors and other RNA-binding proteins, EWSR1 regulates gene expression and coordinates various cellular processes. Its abnormal expressions or mutations are closely linked not only to tumor development but also to the regulation of fibrosis, immune responses, and cellular stress adaptation, highlighting its broad significance in cellular biology. Studies have shown that in cardiac fibrosis, EWSR1 can bind to the mechanosensitive protein VGLL3 [48], inhibiting the expression of miR-29b. MiR-29b is a microRNA that targets collagen mRNA, and its downregulation leads to increased collagen production, a key characteristic of fibrotic tissue. Although this study focuses on cardiac fibrosis, the shared mechanisms of fibrosis across different tissues suggest that EWSR1 may have a similar regulatory role in liver fibrosis. Moreover, EWSR1 has been shown to regulate non-coding RNAs (lncRNAs and miRNAs), which play crucial roles in the pathological process of fibrosis. A study revealed [49] that in glioma, EWSR1 can induce the expression of the circular RNA circNEIL3, which promotes tumor cell proliferation and survival by stabilizing the IGF2BP3 protein. Although this study focuses on tumors, it highlights the important role of EWSR1 in the regulation of non-coding RNAs, a function that may similarly influence cell proliferation, survival, and ECM synthesis during fibrosis.

In conclusion, EWSR1, as a multifunctional RNA/DNA-binding protein, precisely regulates gene transcription, RNA binding, and post-transcriptional processing through its various domains. Additionally, EWSR1 may influence the expression of genes associated with ECM production and cell activation by modulating non-coding RNAs, thereby regulating the progression of fibrosis. Further exploration of the functions of different EWSR1 domains and their alterations under pathological conditions will help uncover its specific mechanisms in fibrosis and other diseases, providing a theoretical basis for developing targeted therapeutic strategies against EWSR1. These findings underscore the role of EWSR1 in transcriptional regulation and RNA metabolism, with its key molecular mechanisms summarized in Table 2.

**Table 2:** EWSR1-mediated transcriptional and non-coding RNA regulatory mechanisms.

Functional Category	Molecular Mechanism	Downstream Effects	Key References
Transcriptional Regulation	Interacts with transcription factors and RNA polymerase II; modulates chromatin accessibility	Promote transcription of target genes, including growth factors, cell cycle regulators, and pro-fibrotic factors	[50,51]
Co-activation in Transcription	Interacts with TATA-binding protein and transcription initiation complexes	Enhances transcription initiation efficiency; stabilizes the transcriptional machinery	[45]
TGF-β/SMAD Pathway Modulation	Binds SMAD3, a key mediator of TGF-β signaling	Regulates HSCs activation and expression of pro-fibrotic genes	[27]
Long Non-Coding RNA Regulation	Modulates expression, stability, and function of lncRNAs	Influences cell differentiation, proliferation, signal transduction, and fibrosis	[45,52]
Circular RNA Regulation	Binds specific circRNAs, regulates back-splicing and stability	Promotes cell proliferation, anti-apoptosis, and ECM synthesis	[49,53]
MicroRNA Regulation	Binds miRNA precursors; influences maturation and function	Modulates HSCs proliferation, apoptosis, and fibrosis-related gene expression	[48,54]
Fibrosis-Specific Effect	Downregulates anti-fibrotic miRNAs (e.g., miR-29b)	Increases collagen production and ECM deposition	[48]
Cross-Disease Regulatory Role	Regulates gene expression via ncRNAs in tumors, cardiovascular, and neurodegenerative diseases	Influences cell proliferation, apoptosis, inflammation, and ECM synthesis	[[55], [56], [57], [58]].

#### 5.1.1 EWSR1 in transcriptional regulation

The role of EWSR1 in transcriptional regulation primarily lies in its interactions with transcription factors and RNA polymerase II [50].

EWSR1 plays a critical role as a chromatin remodeling factor in gene expression regulation, primarily through interactions with transcription factors and RNA polymerase II, modulating the transcriptional activity of specific genes. EWSR1 can directly bind to the activation domains of transcription factors, enhancing their transcriptional activity. For example, in Ewing sarcoma, it forms complexes with the transcriptional co-activators p300 and CBP [51], collaboratively promoting the expression of target genes. This interaction facilitates the acetylation of histones H3 and H4, leading to a relaxation of chromatin structure and increasing the accessibility of transcription factors and RNA polymerase II to DNA, thereby activating the expression of pro-tumor genes. Additionally, EWSR1 can regulate the open state of chromatin through chromatin remodeling complexes and histone acetyl transferases, thereby activating specific gene transcription during processes such as cell differentiation and stress response. This multi-layered regulatory mechanism underscores the crucial role of EWSR1 in gene expression regulation.

In addition, EWSR1 also acts as a co-activator in transcriptional regulation, interacting with RNA polymerase II and the TATA-binding protein (TBP) to facilitate the assembly of the transcription initiation complex [45], ensuring an efficient start to transcription. In this process, EWSR1 collaborates with TBP and the transcription initiation complex (such as TFIID complex) to enhance the stability of the transcription initiation machinery, assisting RNA polymerase II in accurately locating the promoter regions of target genes and stabilizing its binding. This ensures effective initiation of transcription. This mechanism is essential for regulating the expression of fibrosis-related genes, especially under cellular stress conditions, where it helps maintain or modulate the overexpression of these genes. This multi-layered transcriptional regulatory function highlights the critical role of EWSR1 in gene expression and the maintenance of cellular functions.

Meanwhile, the N terminus of EWSR 1 acts as a co-activator to regulate the activity of the promoter after binding with other transcriptional regulatory proteins. EWSR1 participates in the promoter regulation of various genes, particularly those involved in growth factors, cell cycle regulators, and inflammatory mediators. For example, EWSR1 influences cell proliferation and differentiation by regulating the target genes of the Wnt/β-catenin signaling pathway [59]. Under stress conditions, EWSR1 interacts with SMAD3, a key transfuction molecule of TGF-β signaling pathway [27], which is likely closely related to the activation of HSCs during fibrosis. By regulating the expression of TGF-β and other pro-fibrotic factors, EWSR1 could serve as a key target for controlling liver fibrosis.

#### 5.1.2 EWSR1 in non-coding RNA regulation

EWSR1's role in transcriptional regulation also extends to the expression and processing of non-coding RNAs (ncRNAs). Research has shown that EWSR1 can influence the expression of long non-coding RNAs (lncRNAs) and circular RNAs (circRNAs) [45], and it regulates gene expression through interactions with these non-coding RNAs. These lncRNAs play crucial roles in cell development and stress responses, and their dysregulated expression is closely associated with cancer and fibrosis. Moreover, EWSR1's interaction with miRNA precursors suggests it may participate in the processing of specific miRNAs, potentially affecting their maturation and regulatory functions. Through these mechanisms, EWSR1 exerts a broad influence on gene expression, impacting both physiological and pathological processes.

As an RNA-binding protein, EWSR1 plays a crucial role in the regulation of lncRNA expression, stability, and functionality. lncRNAs are essential regulators of cell differentiation, proliferation, and signal transduction pathways, and EWSR1 can interact with specific lncRNAs, influencing their cellular localization, degradation, and interaction with other proteins. Research has shown [60]that in the intestinal epithelial cells of naked mole-rats, even in the absence of FUS, the expression of EWSR1 and TAF15 can partially compensate for the loss of FUS function. This compensatory effect indicates that EWSR1 and TAF15 share functional similarities with FUS, enabling them to interact with NEAT1_2 and support the formation of Paraspeckles. Additionally, the study speculates that FET family proteins might interact with MALAT1, participating in mRNA splicing and post-transcriptional processing, which are critical cellular processes. These findings highlight the significant role of EWSR1 in the lncRNA regulatory network and suggest its extensive influence on gene expression and cellular function maintenance.

EWSR1 plays a crucial regulatory role in the biogenesis and stability of circRNA. CircRNA, a non-coding RNA with a closed-loop structure, is primarily formed through back-splicing of precursor mRNA. EWSR1 can bind to specific circRNAs, modulating their splicing process and influencing their intracellular localization and distribution. Furthermore, EWSR1 serves as an essential component of circRNA-protein complexes, participating in the regulation of cellular processes such as proliferation, anti-apoptosis, and stress responses. Research has shown [49] that EWSR1 regulates the expression of circNEIL3, which stabilizes the IGF2BP3 protein, activates downstream signaling pathways, and promotes cell proliferation and survival. A similar mechanism might exist in the context of fibrosis, where EWSR1 could regulate anti-apoptotic signals in HSCs, maintaining their activated state and contributing to the progression of fibrosis. These findings highlight the multifaceted role of EWSR1 in circRNA regulation, offering new insights into its molecular mechanisms in fibrosis.

EWSR1 participates in the post-transcriptional regulation and stabilization of microRNAs (miRNAs) through direct binding. As a class of small non-coding RNAs, miRNAs primarily regulate gene expression by binding to specific mRNAs, either inhibiting protein translation or accelerating mRNA degradation. EWSR1 can influence the expression and function of specific miRNAs, thereby modulating cell proliferation, apoptosis, and fibrosis-related gene expression.

In cardiac fibrosis, studies have shown [48] that EWSR1 interacts with VGLL3 to suppress the expression of miR-29b. miR-29b is a critical anti-fibrotic miRNA that targets and degrades collagen mRNA, thereby reducing ECM synthesis. When EWSR1 inhibits miR-29b, collagen production increases, exacerbating fibrosis. This regulatory mechanism may also exist in liver fibrosis, where EWSR1 could modulate miR-29b expression to maintain the pro-fibrotic state of HSCs.

As an essential component of the gene expression regulatory network, ncRNAs play a significant role in various pathological processes, including fibrosis, cancer, cardiovascular diseases, and neurodegenerative disorders.

In fibrosis, lncRNAs regulate HSCs activation, apoptosis, and abnormal ECM synthesis through interactions with proteins, DNA, or other RNAs. Researchers have discovered [52] that lncRNA MALAT1 can interact with Smad proteins, SETD2, and PPM1A, forming a complex that regulates the TGF-β/Smad signaling pathway. This promotes the sustained activation of HSCs and enhances the expression of fibrosis-related genes. Such interactions impact the expression of genes associated with liver fibrosis. Additionally, miRNAs also play a crucial regulatory role in fibrosis. Studies have shown [54] that miR-29b expression is downregulated in fibrotic tissues, leading to increased synthesis of type I and type III collagen, exacerbating ECM accumulation. Furthermore, research indicates [53] that CircPSD3 overexpression significantly reduces alpha-smooth muscle actin (α-SMA) expression at both mRNA and protein levels, while simultaneously inhibiting HSCs proliferation and activation.

In tumors, ncRNAs also play a significant role. lncRNA HOTAIR is highly expressed in breast cancer and liver cancer [55,56]. It promotes tumor cell proliferation and metastasis by remodeling chromatin structure and suppressing the expression of tumor suppressor genes. In cardiovascular diseases, previous studies have reported [57] that lncRNA MIAT is upregulated in myocardial infarction patients, promoting cardiomyocyte apoptosis and exacerbating cardiac injury. In neurodegenerative diseases, non-coding RNAs also play a role in disease progression. lncRNA TUG1 is abnormally expressed in neurodeg [58].

Although the pathological mechanisms of fibrosis, tumors, cardiovascular diseases, and neurodegenerative diseases differ, the regulatory roles of ncRNAs in these conditions exhibit similarities. ncRNAs participate in the onset and progression of diseases by influencing cell proliferation, apoptosis, inflammatory responses, and ECM synthesis. Additionally, ncRNAs can serve as biomarkers for disease diagnosis, staging, and prognosis assessment, or as therapeutic targets to improve disease outcomes through specific regulation of their expression.

### 5.2 EWSR1 and its interaction with TGF-β signaling

EWSR1, as an RNA-binding protein, is widely distributed across various tissues and plays a critical role in cell proliferation, differentiation, RNA metabolism, and DNA damage repair. TGF-β, a multifunctional cytokine expressed in almost all cells, participates in numerous cellular biological processes through its signaling pathways, including the regulation of cell growth, proliferation, differentiation, ECM production, and fibrosis. Although both EWSR1 and TGF-β signaling have been independently implicated in fibrotic and tumor-related processes, the precise molecular basis of their interaction in HSCs remains incompletely defined. However, from the perspectives of molecular mechanisms, pathway regulation, and disease relevance, there appears to be a complex and significant relationship between the two.

EWSR1 may influence multiple critical nodes of TGF-β signaling through its RNA-binding ability and protein interactions. The classical TGF-β signaling pathway operates via the Smad-dependent route, in which TGF-β receptors activate R-Smads (Smad2/3), allowing them to form complexes with Co-Smad (Smad4) and translocate to the nucleus to regulate target gene expression. Based on its established roles in RNA binding and transcriptional regulation, EWSR1 may modulate the expression level, stability, or translational efficiency of Smad-related transcripts, thereby indirectly shaping TGF-β/Smad signaling output [17]. In addition, EWSR1 has been reported to interact with Smad3 under specific pathological contexts, suggesting a potential mechanism by which EWSR1 could influence TGF-β–driven transcriptional programs rather than acting as a primary upstream regulator [17].Beyond the classical pathway, TGF-β can also activate various signaling pathways through Smad-independent routes, such as MAPK, PI3K/Akt, JAK/STAT, and Rho-GTPase. Non-classical TGF-β pathways regulate diverse cellular processes, including proliferation, migration, differentiation, and survival, broadening the scope of TGF-β signaling in disease progression [37]. Given that EWSR1 has been implicated in the regulation of signaling networks associated with cell survival and stress adaptation, it is plausible that EWSR1 may indirectly affect non-Smad TGF-β signaling by modulating pathway-associated regulators rather than directly engaging core kinase components [61].

Research has shown that the EWSR1-FLI1 fusion protein disrupts the TGF-β signaling pathway by suppressing the transcription of the TGF-βRII. This suppression reduces cellular sensitivity to TGF-β, thereby promoting tumorigenesis. Mechanistically, EWSR1-FLI1 binds directly to the TGF-βRII promoter, inhibiting its transcriptional activity and impairing downstream TGF-β signaling. This loss of TGF-β signaling is considered a critical step in the progression of Ewing sarcoma [62]. It should be noted that these findings were derived from Ewing sarcoma models involving the oncogenic EWSR1-FLI1 fusion protein rather than wild-type EWSR1 and therefore reflect a disease-specific transcriptional mechanism rather than a universal mode of EWSR1 action. Nevertheless, this study provides important conceptual evidence that EWSR1-related proteins are capable of directly modulating TGF-β receptor–level transcriptional control, supporting the biological plausibility that EWSR1 may influence TGF-β signaling through transcriptional mechanisms in other pathological contexts, including fibrosis.

There is a new research that shows that EWSR1 plays a significant role in hepatic fibrosis by influencing fibrogenic gene expression and the activation of HSCs. A recent study reported that EWSR1 overexpression in LX-2 cells was associated with increased mRNA and protein expression of fibrosis-related markers, including collagen type I alpha 1 chain (COL1A1), α-SMA, and TGF-β1 [17]. Conversely, EWSR1 knockdown attenuated the expression of these profibrotic markers, supporting a potential regulatory role of EWSR1 in hepatic fibrogenic responses rather than establishing direct causality. The study further reported that modulation of EWSR1 expression was associated with corresponding changes in connective tissue growth factor (CTGF) levels, a downstream effector of TGF-β1 signaling involved in ECM production, supporting a potential link between EWSR1 and TGF-β–related fibrogenic pathways. In this context, the study suggested that EWSR1 may influence TGF-β–related gene expression partly through post-transcriptional mechanisms involving microRNA-associated mRNA stabilization, although the precise molecular intermediates remain to be fully elucidated. Collectively, these observations indicate that EWSR1 may participate in TGF-β–associated profibrotic signaling in activated HSCs, supporting its consideration as a candidate regulatory factor rather than a validated therapeutic target at the current stage.

In fibrotic diseases, TGF-β is the core driver of fibrosis, and its hyperactivation leads to an abnormal accumulation of collagen and the ECM. In the progression of fibrosis, the phosphorylation and nuclear translocation of SMAD3 are important steps in TGF-β signaling, directly promoting the expression of fibrosis genes (such as COL1A1 and FN 1). Recent evidence suggests that EWSR1 may influence HSC activation and fibrogenic gene expression by modulating components downstream of TGF-β signaling, rather than acting as a primary initiator of the fibrotic cascade. Consistent with its established RNA-binding and transcriptional regulatory properties, EWSR1 has been reported to interact with SMAD3 under specific pathological contexts, supporting the possibility that EWSR1 may modulate SMAD3-dependent transcriptional programs associated with fibrogenesis. Taken together, current evidence supports a model in which EWSR1 functions as a context-dependent regulatory factor that fine-tunes TGF-β/SMAD-associated transcriptional output in activated HSCs, while the precise molecular hierarchy and causal relationships remain to be further elucidated.

### 5.3 Relationship between EWSR1 and oxidative stress

#### 5.3.1 Role of EWSR1 in stress- granule formation

EWSR1 plays a key role in the formation and regulation of stress granules (SGs). These granules are dynamic ribonucleoprotein complexes rapidly assembled by cells in response to environmental stressors such as oxidative stress, heat shock, viral infections, or toxic damage. They primarily consist of untranslated mRNA, RNA-binding proteins, non-RNA-binding proteins, and translation factors [63]. By temporarily suppressing the translation of specific mRNAs and storing them, SGs reduce the cell's metabolic burden, enabling it to better adapt to adverse conditions. In this context, EWSR1 has been implicated as a component of SGs that participates in stress-adaptive responses, although its precise regulatory hierarchy within SGs assembly remains incompletely defined.

Under stress conditions, EWSR1, an RNA-binding protein, relocates from the nucleus to the cytoplasm, where it actively participates in SGs formation. Utilizing its RRM, EWSR1 binds directly to untranslated mRNA and interacts with other RNA-binding proteins [64], including TIA-1, G3BP1, and FUS, assembling into large ribonucleoprotein complexes essential for SGs stability and function. EWSR1 interacts not only with mRNA but also with dissociated translation factors, such as eIF4G and eIF3 [63], thereby contributing to translational repression during stress conditions. Additionally, SGs temporarily store specific mRNAs, which can be rapidly re-released for translation once the stress subsides, facilitating efficient recovery and cellular repair. Through these interactions, stress granule assembly is associated with transient translational repression and cellular stress adaptation, rather than serving as a direct determinant of long-term cell fate.

During the stress response, the large molecular complexes formed by EWSR1 and RNA-binding proteins are essential for the assembly of SGs. Acting as a scaffold molecule, EWSR1 facilitates the aggregation of RNA-protein complexes. The low-complexity domain (LCD) of EWSR1 plays a critical role in its scaffolding function, particularly in the formation of SGs. This domain mediates liquid-liquid phase separation (LLPS) [65,66], a key mechanism for the rapid assembly of membrane less organelles. Through LLPS, EWSR1 facilitates the quick aggregation of untranslated mRNA and associated proteins, forming dynamic stress granule structures. LLPS-mediated assembly enables SGs to form and dissolve dynamically in response to fluctuating stress conditions, supporting reversible cellular adaptation rather than irreversible phenotypic commitment. In fibrotic microenvironments characterized by persistent oxidative stress, prolonged or dysregulated SGs dynamics have been proposed to support stress tolerance in activated cells, including HSCs, although direct causal evidence in liver fibrosis remains limited.

The scaffolding function of EWSR1 not only facilitates the stable assembly of SGs but also orchestrates the orderly recruitment of proteins and mRNA molecules, finely regulating the composition and function of SGs. This organized assembly and regulation ensure that SGs can rapidly respond to cellular stress and efficiently disassemble once the stress is relieved, maintaining cellular homeostasis. However, when EWSR1 undergoes genetic mutations or abnormal localization, it may lead to aberrant aggregation or delayed disassembly of SGs, impairing the cell's ability to adapt to environmental stress and potentially contributing to pathological conditions such as neurodegenerative diseases, fibrosis, and cancer. In neurodegenerative diseases, such as amyotrophic lateral sclerosis (ALS) and frontotemporal dementia (FTD), abnormal aggregation of EWSR1 with proteins like FUS leads to the persistent presence of SGs, disrupting normal neuronal function [67]. EWSR1 dysfunction is also associated with fibrosis and cancer. In fibrotic microenvironments characterized by persistent oxidative stress, prolonged or dysregulated SGs dynamics have been proposed to support stress tolerance in activated cells, but direct causal evidence specifically linking SGs alterations to HSC persistence and ECM accumulation in liver fibrosis remains limited [68].

#### 5.3.2 Regulation of EWSR1 by oxidative stress

**Fig. 3:**
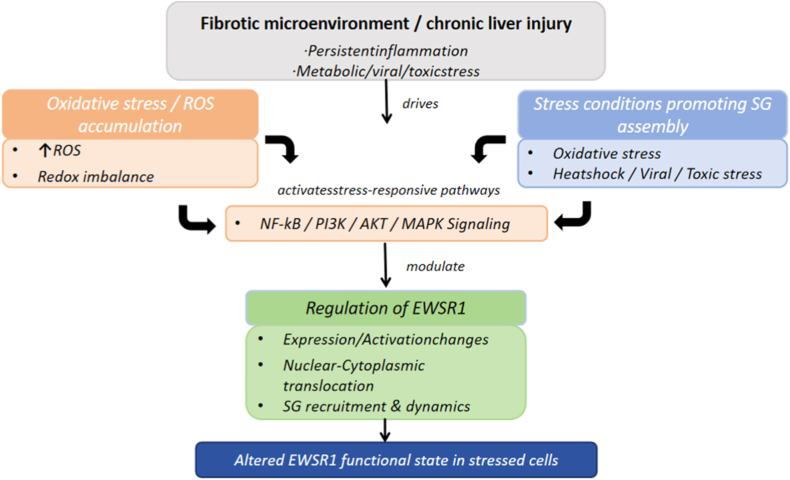
**Upstream regulatory mechanisms influencing EWSR1 under oxidative stress in fibrotic microenvironments.** Chronic liver injury and fibrotic microenvironments are characterized by persistent oxidative stress and ROS accumulation. These stress cues activate canonical stress-responsive pathways, including NF-κB, PI3K/AKT, and MAPK signaling, which have been implicated in regulating EWSR1 expression/activity, nuclear–cytoplasmic redistribution, and SGs recruitment and dynamics. This schematic summarizes the upstream regulatory context shaping the functional state of EWSR1 during cellular stress.

Oxidative stress plays a critical role in regulating EWSR1, serving as an essential mechanism for cellular adaptation to injury and stress. EWSR1 is involved in key processes such as RNA metabolism, transcriptional regulation, and SGs formation. As oxidative stress drives profibrotic and proinflammatory responses, it can significantly influence EWSR1's expression, subcellular localization, and functional activity. These regulatory effects are closely linked to the progression of various diseases, including liver fibrosis, neurodegenerative disorders, and cancer. The upstream regulatory context through which oxidative stress and stress-responsive signaling pathways modulate EWSR1 expression, localization, and SGs dynamics is summarized in Fig. 3.

Under oxidative stress, elevated intracellular ROS trigger a series of molecular and cellular alterations, significantly impacting EWSR1's function. ROS can influence EWSR1 at multiple levels, including its transcriptional expression, nuclear-cytoplasmic distribution, and its role in the assembly and disassembly of SGs. Through post-translational modifications, oxidative stress can fine-tune EWSR1's involvement in RNA transcription and splicing, contributing to cellular stress responses and repair mechanisms.

Oxidative stress can activate multiple cellular signaling pathways, including NF-κB, PI3K/AKT, and MAPK [69], which in turn modulate the expression and activity of EWSR1 to enhance cellular stress responses. ROS can upregulate EWSR1 expression or promote its activation, thereby strengthening its transcriptional regulatory capacity and influencing stress-related gene expression. Additionally, oxidative stress-induced epigenetic modifications, such as acetylation, methylation, and phosphorylation [70], can alter EWSR1's transcriptional activity and its interactions with other proteins, further fine-tuning its role in cellular stress defense mechanisms. Collectively, these signaling and epigenetic mechanisms suggest that oxidative stress regulates EWSR1 through multiple convergent pathways; however, the relative contribution of each pathway may vary depending on cell type and pathological context [69,70].

Oxidative stress significantly alters the intracellular distribution of EWSR1. Under normal conditions, EWSR1 primarily resides in the nucleus, where it regulates gene transcription and RNA processing [66]. However, in response to oxidative stress, EWSR1 relocates to the cytoplasm and actively participates in SGs assembly. This redistribution represents an adaptive mechanism to limit metabolic demand and preserve RNA integrity during stress. Stress granule formation is a vital adaptive mechanism for managing oxidative stress, and EWSR1 has been identified as an important structural and regulatory component involved in stress granule dynamics. By interacting with key RNA-binding proteins such as TIA-1 and G3BP1, EWSR1 facilitates the aggregation of RNA-protein complexes, supporting the efficient assembly and stability of SGs [68]. These granules dissolve rapidly after stress relief, restoring normal RNA metabolism. However, EWSR1 function may be inhibited or disrupted under conditions of persistent or excessive oxidative stress. For example, SGs may fail to dissolve properly, resulting in long-term RNA accumulation and translational repression. This phenomenon is particularly prominent in certain diseases, such as neurodegenerative diseases (such as ALS) and chronic inflammatory diseases.Abnormal localization of EWSR1 can disrupt cell cycle regulation and proliferation signaling pathways, potentially triggering uncontrolled cell growth and contributing to tumor initiation and progression. Taken together, the transition from reversible stress granule assembly to persistent dysregulation under chronic oxidative stress suggests a shift from adaptive RNA protection toward pathological RNA metabolism, although direct causal links in liver fibrosis remain to be fully established.

The regulation of EWSR1 by oxidative stress may also affect the development of liver fibrosis through an interaction with TGF-β signaling. In the fibrotic microenvironment, ROS can not only promote the expression of TGF-β signaling but may also participate in the expression of liver fibrosis-related genes, possibly through the regulation of EWSR1. The sustained activation of HSCs depends on the positive feedback loop of oxidative stress and TGF-β signaling, and the altered function of EWSR1 may exacerbate the maintenance of this feedback loop and further worsen liver fibrosis. At present, evidence supporting EWSR1 as a mediator linking oxidative stress to TGF-β–driven fibrotic signaling is largely indirect, and this relationship should be interpreted as a putative regulatory axis rather than a fully established causal pathway [18,26].

EWSR1 may also directly influence HSC survival by regulating apoptosis-related genes. In the fibrotic environment, HSCs exhibit significant anti-apoptotic capability, primarily regulated by the Bcl-2 protein family, where the balance between anti-apoptotic proteins (such as Bcl-2) and pro-apoptotic proteins (such as Bax) is crucial [71]. If EWSR1 suppresses anti-apoptotic signaling while enhancing pro-apoptotic pathways, this shift may disrupt the survival advantage of activated HSCs and facilitate mitochondria-dependent apoptosis. Additionally, EWSR1 may influence HSC anti-apoptotic capacity through modulation of the PI3K/AKT pathway, a central pro-survival signaling axis that promotes anti-apoptotic protein expression and suppresses apoptotic execution programs in activated HSCs. Whether inhibition of EWSR1 attenuates PI3K/AKT signaling and thereby sensitizes activated HSCs to apoptosis remains to be determined and represents an important direction for future mechanistic investigation.

Beyond classical signaling cascades, emerging gene-editing studies highlight the intricate relationship between metabolic regulation and apoptosis control. For instance, CRISPR/Cas9-mediated disruption of purine transport pathways has been shown to induce mitochondrial apoptosis and alter cellular metabolic homeostasis in colorectal models [72]. These findings emphasize the interconnectedness between metabolic signaling and programmed cell death, a concept that may hold mechanistic relevance for understanding HSC survival and apoptosis resistance in fibrotic conditions.

To verify whether EWSR1 affects HSC apoptosis by regulating ROS production or the antioxidant system, integrated experimental approaches using both cellular and animal models are required. By knocking down EWSR1 or treating cells with its inhibitor, changes in ROS levels, as well as the expression and activity of antioxidant enzymes in HSCs, can be assessed to elucidate its role in oxidative stress. Meanwhile, evaluating the expression of apoptosis-related genes (such as Bcl-2, Bax, and Caspase-3), combined with apoptosis rate analysis, will further clarify the regulatory role of EWSR1 in HSC apoptosis. Collectively, these experimental strategies provide a framework for delineating the role of EWSR1 in oxidative stress–associated apoptosis during liver fibrogenesis, as summarized in Table 3.

**Table 3:** Functional roles of EWSR1 in TGF-β signaling, oxidative stress, and apoptosis in HSCs.

Function/Role	Target/Mechanism	Experimental Model/Methods	Findings/Effects	References
Modulation of TGF-β signaling	Binding to Smad mRNA; interaction with Smad3 protein; regulation of TGF-β1, COL1A1, α-SMA, CTGF	LX-2 cells (HSC line), overexpression and knockdown experiments, qPCR, Western blot	Overexpression increases fibrosis markers; knockdown decreases markers; stabilizes mRNA of fibrosis-related genes; promotes HSC activation	[17,62].
Interaction with non-classical TGF-β pathways	Modulation of PI3K/Akt, MAPK, JAK/STAT	Cell signaling assays	Enhances HSCs migration; promotes anti-apoptotic effects	[37,61]
Stress granule formation under oxidative stress	Scaffolding via RRM and low-complexity domain (LCD); interaction with TIA-1, G3BP1, FUS; LLPS-mediated assembly	Stress-induced LX-2 cells, fluorescence imaging	Promotes SGs assembly; inhibits translation temporarily; conserves energy; protects RNA; maintains cellular homeostasis	[63,65,66],
Regulation under oxidative stress	Nuclear-cytoplasmic translocation; post-translational modifications; interaction with NF-κB, PI3K/AKT, MAPK	ROS treatment in HSCs, Western blot, immunofluorescence	Enhances transcriptional regulatory capacity; modulates stress-related gene expression; maintains adaptive response	[69,70]
Regulation of apoptosis in HSCs	Modulation of Bcl-2, Bax, Caspase-3; inhibition of PI3K/AKT	LX-2 cells and fibrotic mouse models; apoptosis assays (flow cytometry, TUNEL)	Promotes apoptosis by downregulating anti-apoptotic proteins and upregulating pro-apoptotic proteins; weakens PI3K/AKT survival signaling	[66]

### 5.4 The potential role of EWSR 1 in fibrosis

As an RNA-binding protein, EWSR1 plays a key role in gene expression, RNA splicing, and cellular stress responses. RNA-binding proteins (RBPs) are a class of proteins that regulate various RNA processes, including generation, splicing, maturation, transport, translation, and degradation, by recognizing specific RNA sequences or structural elements. Serving as essential mediators in post-transcriptional regulation, RBPs bridge the gap between gene transcription and functional protein translation. The specificity and efficiency of RNA binding by RBPs depend on their RNA-binding domains (RBDs). These domains include the RRM, which utilizes α-helices and β-sheets to directly interact with RNA; the zinc-finger domain, stabilized by zinc ions to facilitate RNA binding; the double-stranded RNA-binding domain (dsRBD), specialized for binding double-stranded RNA; and DEAD-box helicase domains, which are involved in RNA metabolism through their helicase activity. Additionally, many RBPs possess multifunctional domains, allowing them to interact simultaneously with RNA and proteins, enabling their integration into larger protein complexes and participation in diverse cellular regulatory networks. Within this regulatory framework, EWSR1 represents a multifunctional RBP whose structural composition and interaction capacity suggests a potential role in coordinating RNA metabolism with stress-adaptive and profibrotic cellular responses.

Abnormal expression and dysregulation of RBPs are closely associated with the progression of multiple pathological conditions, including fibrosis. In liver fibrosis, RBMS3 has been reported to be significantly upregulated in activated HSCs. RBMS3 binds to specific sites within the 3′ untranslated region (3′ UTR) of Prx1 mRNA, thereby enhancing its stability and promoting increased protein synthesis. Because PRX1 functions as a transcriptional activator of the collagen α1(I) promoter, this post-transcriptional regulation reinforces the profibrotic phenotype of activated HSCs and contributes to fibrosis progression [73]. Additionally, the RBP ELAVL1/HuR has been shown to modulate ferroptosis in HSCs through activation of ferritinophagy, a process that may promote HSC apoptosis and attenuate fibrotic responses [74]. Experimental evidence indicates that exposure to ferroptosis-inducing stimuli results in elevated ELAVL1 protein levels, mediated in part by suppression of ubiquitin–proteasome–dependent degradation. Conversely, siRNA-mediated knockdown of ELAVL1 confers resistance to ferroptosis, whereas ELAVL1 overexpression enhances classical ferroptotic features. These observations, derived from both in vitro HSC models and primary HSCs isolated from hepatocellular carcinoma–associated fibrotic liver tissues, underscore the functional relevance of RBPs in regulating cell fate decisions during fibrogenesis.

Increasing evidence indicates that RBPs are themselves subject to regulation by non-coding RNAs, particularly long lncRNAs. In the context of liver fibrosis, Wu et al. reported that linc-SCRG1 accelerates fibrogenesis by suppressing the RNA-binding protein TTP [75]. In activated LX-2 cells, overexpression of TTP leads to downregulation of linc-SCRG1 and promotes degradation of its downstream profibrotic targets, including MMP-2 and TNF-α, thereby exerting an antifibrotic effect. Beyond liver fibrosis, dysregulated expression or functional impairment of RBPs has been extensively implicated in a wide spectrum of pathological conditions, most notably cancer. RBPs regulate multiple cancer-associated cellular phenotypes, including proliferation, apoptosis, senescence, migration, invasion, and angiogenesis, thereby influencing tumor initiation, progression, and clinical outcome. For instance, members of the cytoplasmic polyadenylation element binding (CPEB) protein family are evolutionarily conserved RBPs that function as critical regulators of mRNA transport and translation and have been implicated in cancer development and progression [76]. Similarly, RBP dysfunction is a hallmark of several neurodegenerative diseases. Proteins such as TDP-43 and FUS exhibit aberrant aggregation and impaired RNA metabolism in ALS and FTD, resulting in toxic RNA–protein complexes and neuronal dysfunction [77]. In cardiovascular disease, RBPs also contribute to pathological remodeling processes. For example, Quaking (QKI) regulates cardiac fibroblast activation and thereby modulates the extent of cardiac fibrosis [78]. In metabolic disorders, RBPs have been shown to influence insulin sensitivity and lipid metabolism by controlling the stability and translation of mRNAs involved in insulin signaling pathways [79]. Collectively, these findings across diverse disease contexts highlight RBPs as central regulators of cell fate decisions and stress-adaptive responses, providing a conceptual framework for considering EWSR1 as a potential modulator of fibrotic processes.

Research directly examining the relationship between EWSR1 and liver fibrosis remains limited. Nevertheless, TGF-β/SMAD signaling is well established as a central driver of HSC activation. Upon activation, TGF-β initiates canonical SMAD-dependent signaling through binding to TGF-βRII, leading to induction of downstream fibrotic genes, including type I and III collagens (COL1A1 and COL3A1) and FN1. In parallel, TGF-β reinforces the profibrotic phenotype of HSCs through non-SMAD pathways, such as PI3K/AKT and MAPK signaling, thereby enhancing proliferation, migration, and resistance to apoptosis. Within this established signaling framework, EWSR1 has been proposed as a potential modulator of key regulatory nodes, rather than a primary driver, based on its RNA-binding and transcriptional regulatory capacities. From a mechanistic perspective, the RNA-binding and transcriptional regulatory functions of EWSR1 provide multiple avenues through which it could influence TGF-β/SMAD signaling. EWSR1 may regulate the expression or stability of key components within the TGF-β pathway at the transcriptional or post-transcriptional level. Notably, EWSR1–SMAD3 fusion events have been reported in fibroblastic tumors, supporting a functional interaction between these molecules [27]. SMAD3 itself is a critical intracellular mediator of TGF-β signaling and a key regulator of ECM synthesis in fibroblasts. These observations suggest a mechanistic link between EWSR1 and TGF-β signaling components, although direct evidence delineating this interaction in HSCs during liver fibrosis remains to be fully established.

Bao et al. were the first to directly implicate EWSR1 in HSC activation and liver fibrosis. In their study, the small-molecule compound 6K was shown to bind EWSR1 and suppress HSC activation, leading to reduced expression of fibrosis-associated genes and attenuation of fibrotic progression [17]. Notably, this work represents the earliest experimental evidence positioning EWSR1 as a candidate therapeutic target in liver fibrosis, extending its previously established relevance beyond oncogenic contexts. Although these findings provide proof-of-concept support for EWSR1-directed antifibrotic intervention, further studies are required to clarify the specificity, mechanism of action, and long-term safety of EWSR1 inhibition in fibrotic liver disease.

EWSR1 has been reported to regulate the expression of multiple apoptosis-related genes, including the anti-apoptotic factor Bcl-2 and the pro-apoptotic protein Bax [80]. In this context, modulation of EWSR1 activity may alter the apoptotic balance within activated HSCs by reducing Bcl-2 expression and enhancing pro-apoptotic signaling, thereby increasing susceptibility to cell death. In parallel, EWSR1 inhibition has been proposed to attenuate TGF-β–induced prosurvival pathways, such as PI3K/AKT signaling, which are known to reinforce apoptosis resistance in activated HSCs [61]. Together, these pathways converge on apoptotic regulation rather than representing independent mechanisms.

In addition to direct effects on apoptotic gene expression, EWSR1 may influence TGF-β signal transduction by modulating endogenous negative feedback regulators such as SMAD7. SMAD7 competitively binds to TGF-βRI, preventing phosphorylation of SMAD2/3 and thereby dampening downstream profibrotic signaling. If suppression of EWSR1 leads to upregulation of SMAD7, as suggested by its transcriptional regulatory capacity, this mechanism could further constrain TGF-β–driven HSC activation.

Furthermore, EWSR1 inhibition may impact non-SMAD signaling cascades downstream of TGF-β. Experimental evidence suggests that EWSR1 can indirectly modulate PI3K/AKT signaling, a pathway that not only intersects with TGF-β signaling but also directly governs HSC proliferation and apoptosis resistance [61]. By reducing AKT activity, EWSR1 inhibition may diminish prosurvival signaling and sensitize HSCs to apoptotic cues. Concomitant effects on MAPK pathways, including ERK, JNK, and p38, have also been proposed, which may collectively restrain HSC migration and profibrotic behavior.

Taken together, current evidence supports a model in which EWSR1 functions as a multifunctional regulatory node integrating apoptotic control, TGF-β–associated signaling, and stress-adaptive pathways in activated HSCs, rather than as a single linear effector. Nevertheless, rigorous validation using both in vitro and in vivo fibrotic models is required to delineate cell-type specificity, downstream targets, and long-term safety considerations associated with EWSR1-targeted intervention. These challenges underscore both the translational promise and the therapeutic complexity of targeting EWSR1 in liver fibrosis, highlighting the need for pathway-selective or context-dependent strategies in future antifibrotic drug development.

## 6 Future perspectives

Accumulating evidence suggests that EWSR1 functions as an important regulatory node linking profibrotic signaling, oxidative stress responses, and apoptosis resistance in HSCs. While current findings highlight its potential relevance in liver fibrogenesis, several key issues remain to be addressed before EWSR1 can be considered a viable therapeutic target. First, most available evidence regarding EWSR1 function in HSCs is derived from in vitro models or correlative analyses, and in vivo validation using well-characterized fibrosis models will be essential to establish its functional significance under physiological conditions. In particular, tissue-specific manipulation of EWSR1 expression may help clarify its cell-type–dependent roles within the complex hepatic microenvironment.

Second, given that EWSR1 is a multifunctional RNA-binding protein involved in transcriptional regulation, RNA metabolism, and stress granule dynamics across diverse tissues, therapeutic strategies targeting EWSR1 must carefully consider specificity and safety. Systemic inhibition of EWSR1 may lead to unintended effects on genome stability, stress responses, or normal cellular homeostasis. Future efforts may therefore focus on selectively modulating EWSR1 activity in activated HSCs or disrupting specific EWSR1-mediated interactions that are preferentially engaged during fibrogenesis, rather than globally suppressing its expression.

In addition, the potential involvement of EWSR1 in the transition from liver fibrosis to hepatocellular carcinoma (HCC) warrants further investigation. Chronic fibrosis provides a permissive microenvironment for malignant transformation, and EWSR1 has been implicated in oncogenic processes through its roles in transcriptional control and RNA regulation. Whether sustained dysregulation of EWSR1 contributes to fibrosis-associated carcinogenesis or represents a molecular link between fibrotic remodeling and tumor initiation, remains an open question that merits systematic exploration.

Finally, advances in RNA biology and targeted delivery technologies may provide new opportunities to exploit EWSR1-related pathways for antifibrotic intervention. Approaches such as RNA-based therapeutics, small molecules targeting protein–RNA interactions, or strategies aimed at modulating stress-responsive signaling upstream of EWSR1 could offer alternative routes to attenuate fibrogenic signaling while minimizing off-target effects. Together, these future directions underscore the need for integrated mechanistic and translational studies to fully define the therapeutic potential of EWSR1 in liver fibrosis.

## 7 Conclusion

EWSR1 has emerged as a potentially important regulatory factor in HSC biology through its involvement in TGF-β–associated signaling, oxidative stress responses, and apoptosis-related pathways. By integrating transcriptional and post-transcriptional regulatory mechanisms, EWSR1 influences fibrogenic gene expression, stress granule dynamics, and cell survival programs that are central to HSC activation and persistence during liver fibrosis. These findings position EWSR1 as a novel molecular node within the complex regulatory network governing fibrogenesis.

However, it is important to emphasize that current evidence supporting the role of EWSR1 in liver fibrosis remains largely derived from in vitro studies and correlative observations. The precise molecular mechanisms by which EWSR1 coordinates profibrotic signaling and stress adaptation in vivo, as well as its cell-type–specific functions within the hepatic microenvironment, require further investigation. Moreover, given the broad biological roles of EWSR1 across multiple tissues, therapeutic targeting of this RNA-binding protein will necessitate careful consideration of specificity, safety, and long-term consequences.

In this context, future studies aimed at dissecting the upstream regulatory cues controlling EWSR1 activity, its downstream effector pathways, and its potential involvement in fibrosis-associated HCC will be critical. Together, a more comprehensive understanding of EWSR1 biology may facilitate the development of refined antifibrotic strategies that selectively disrupt pathogenic signaling while preserving essential cellular functions.

## Informed consent statement

Not applicable.

## Institutional review board statement

Not applicable.

## Declaration of AI-assisted writing

During the preparation of this work, the authors used an AI-assisted language model (ChatGPT, OpenAI) to support language editing, structural refinement. After using this tool, the authors reviewed and edited the content as needed and take full responsibility for the integrity and accuracy of the manuscript.

## Funding

Not applicable.

## CRediT authorship contribution statement

**Zhang Shiwan:** Data curation, Formal analysis, Investigation, Methodology, Writing – original draft. **Luo Guangcheng:** Conceptualization, Funding acquisition, Resources, Supervision. **Maisarah Abdul Mutalib:** Conceptualization, Funding acquisition, Project administration, Supervision, Validation.

## Declaration of competing interest

The authors declare that they have no known competing financial interests or personal relationships that could have appeared to influence the work reported in this paper.

## Data Availability

No data was used for the research described in the article.
